# Constructing melanopic light exposure profiles from sparse daily-route measurements

**DOI:** 10.1038/s44323-026-00108-x

**Published:** 2026-10-16

**Authors:** Niloufar Tabandeh, Jennifer A. Veitch, Manuel Spitschan

**Affiliations:** 1 Chronobiology & Health, TUM School of Medicine and Health, Technical University of Munich, Munich, Germany; 2 Translational Sensory & Circadian Neuroscience, Max Planck Institute for Biological Cybernetics, Tübingen, Germany; 3 Construction Research Centre, National Research Council of Canada, Ottawa, ON Canada; 4 TUM Institute for Advanced Study (TUM-IAS), Technical University of Munich, Garching, Germany; 5 TUMCREATE Ltd., Singapore, Singapore

**Keywords:** Ecology, Ecology, Environmental sciences

## Abstract

Daily light exposure is temporally structured, yet calibrated spectral measurements are usually available only at selected locations and times. Turning such a sparse library into a schedule-resolved profile requires an assignment rule: a decision about which measurements may represent each unmeasured part of the day. We examined how much that decision matters. Sixty-three eye-level spectral irradiance measurements, collected across home, commute, office and lunch settings on three June days in Ottawa, were mapped onto a schedule of 22 half-hour slots. For each of six assignment rules we generated 100,000 profiles and computed melanopic dose and time above thresholds of 250, 1,000 and 10,000 melanopic lx. Indoor-only and outdoor-only assignments gave contrasting bounds, with median doses of 16,536 and 119,995 lx ⋅ h. Among the four rules that combined indoor and outdoor measurements, median dose varied only from 44,257 to 48,337 lx ⋅ h, whereas the central 95% of the generated dose distributions spanned ranges differing by nearly a factor of two. Central estimates were set largely by the composition of the eligible measurement pools rather than by the structure a rule preserved; the rule mainly determined spread. Targeted substitutions, in which only commute and lunch conditions were changed, raised median dose by 28,600 lx ⋅ h, with outdoor lunch contributing more cumulative dose and outdoor commuting more time above 10,000 lx. The framework supports sensitivity and scenario analysis rather than reconstruction of continuous personal exposure, and shows why the assignment step must be reported explicitly for a constructed profile to be interpretable.

## Introduction

Light exposure changes as people move between indoor and outdoor environments across the day. The resulting pattern can affect circadian timing, sleep, alertness and neuroendocrine physiology through retinal pathways that include melanopsin-expressing intrinsically photosensitive retinal ganglion cells^1–5^. These responses depend on the spectrum, timing and duration of exposure, not only on light intensity at a single moment. The CIE melanopic equivalent daylight illuminance (melanopic EDI) provides a standardised measure of melanopsin-weighted stimulation^4,6^. A daytime melanopic EDI of at least 250 lx has been recommended for healthy adults^7^, but a single measurement cannot show whether that level was sustained or whether the day contained shorter periods of much brighter light. It is therefore common to use two complementary summaries: melanopic dose, which integrates exposure over represented time, and time above threshold (TAT), which quantifies how long exposure exceeded a specified level.

Continuous wearable light logging is the most direct approach for characterising an individual’s daily light exposure. Its output nevertheless depends on the device, spectral calibration, wearing position, orientation, body shading, compliance and processing decisions^8–12^. Continuous monitoring may also be impractical or unavailable. Light conditions may need to be assessed before participants occupy a building or follow a planned route; body-worn devices may be unsuitable in some clinical, industrial, educational or security-sensitive settings; and existing environmental measurements may have no accompanying wearable data. In these situations, repeated measurements at selected locations and times may provide the most feasible basis for describing potential exposure across a daily routine.

Calibrated eye-level spectroradiometry characterises the spectrum and magnitude of light at a defined location and viewing geometry, but only at the times it is used. Measurements from homes, offices, commutes and outdoor spaces therefore form a library of observed conditions rather than a complete daily time series. If the sequence and approximate duration of activities are known, that library can be assembled into schedule-resolved profiles, which raises the central methodological question: how should observed measurements represent the unmeasured parts of the schedule?

Related approaches have been developed in architectural lighting and environmental exposure science. Climate-based lighting models construct time-resolved eye-level exposure estimates from discrete spatial and temporal simulations and use them to compare alternative building and lighting scenarios^13–15^. More recently, behavioural trajectories, spatial context and environmental information have been combined to reconstruct individual melanopic light exposure without wearable light sensing at deployment^16^. In environmental exposure science, microenvironment models combine time-activity schedules with setting-specific environmental estimates, while stochastic diary-assembly methods construct longitudinal exposure sequences from sparse observations^17,18^. These approaches share the principle that environmental information can be organised using an explicit temporal structure. Less attention has been given to the assignment step itself: how alternative rules for placing sparse measured light conditions into a daily schedule affect the resulting exposure summaries.

Consider a half-hour office slot at 14:00. It could be represented by any measurement in the library, only measurements recorded in the same half-hour bin, any office measurement, or only measurements from an afternoon-office block. Each choice preserves different information about clock time, location and activity context. We refer to these choices as *assignment rules*. Once a rule is specified, repeated sampling from the eligible measurements produces a distribution of constructed profiles and corresponding distributions of melanopic dose and TAT. Their centre, spread and extremes depend both on variation among the measured environments and on the assumptions used to assemble them. Comparing plausible rules therefore provides a sensitivity analysis of the constructed exposure estimates.

The same framework supports targeted scenario substitutions: outdoor walking measurements can replace in-car commute measurements, or outdoor lunch can replace indoor lunch, while the rest of the schedule is held fixed. Such comparisons show how exposure summaries change when one measured condition is substituted for another within a defined routine; they are not causal estimates of behavioural change.

The aims of this study were to (1) state transparent rules for constructing schedule-resolved melanopic light exposure profiles from sparse measurements, (2) determine which features of the resulting dose and TAT distributions are sensitive to the choice of rule and which are not, and (3) demonstrate targeted substitutions for commute and lunch conditions. We addressed these aims using 63 calibrated eye-level spectral irradiance measurements collected along a predefined home-commute-office route on three non-consecutive days, represented as 22 ordered half-hour slots.

## Results

### Outdoor measurements created distinct peaks in the observed route days

**Fig. 1:**
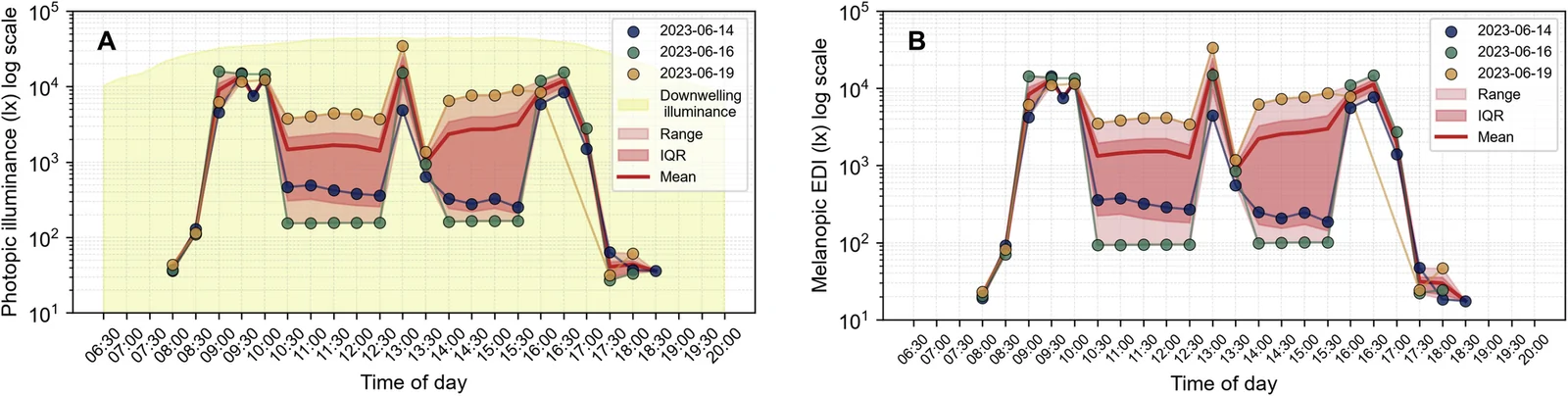
Light exposure measured along the daily route on three days. **A** Photopic illuminance as a function of time of day (log scale), including average downwelling photopic illuminance from rooftop measurements. **B** Melanopic EDI (log scale). In both panels, the solid line shows the mean across available observations at each time, and shaded regions indicate the interquartile range and full range.

The three measurement days followed the same broad sequence of home, commute, office, lunch, return commute and home. Outdoor commute and lunch measurements produced the highest photopic illuminance and melanopic EDI values, whereas home and office measurements were generally lower (Fig. 1). The office with electric lighting only remained low and relatively stable, while the two daylit offices reached higher values and varied more across the day. This indoor-outdoor contrast matters for the analyses below because each assignment rule controls when the bright outdoor measurements may enter a constructed profile.

The observed days did not contain the same number of measurements. They comprised 23, 21 and 19 half-hour epochs, representing 11.5, 10.5 and 9.5 h, respectively (Table 1). Across the three day-level summaries, mean melanopic dose was 44,508 lx ⋅ h. Mean TAT was 6.2 h above 250 lx, 5.0 h above 1000 lx and 1.8 h above 10,000 lx. These values describe the epochs sampled on each date. Because the measured durations were unequal and did not provide continuous coverage, they are shown as contextual reference summaries rather than as ground truth for validating the generated profiles.

**Table 1:** Melanopic summary metrics across the three observed daily-route measurement days

Date	Epochs	Represented time (h)	Dose (lx ⋅ h)	TAT > 250 lx (h)	TAT > 1000 lx (h)	TAT > 10,000 lx (h)
2023-06-14	23	11.5	29,941	7.0	4.0	1.0
2023-06-16	21	10.5	43,342	4.0	3.5	3.0
2023-06-19	19	9.5	60,243	7.5	7.5	1.5
Mean	21	10.5	44,508	6.2	5.0	1.8

Each observation represented a 30-min epoch, so represented time is the number of available epochs multiplied by 0.5 h. Melanopic EDI thresholds are reported in lx and dose in lx ⋅ h.

### Six assignment rules encoded different assumptions about representativeness

We defined six assignment rules that differed only in which measurements were eligible to fill each of the 22 half-hour schedule slots. The rules ranged from unrestricted pooling to matching by clock time or activity context. Each rule therefore answered a different question about which observed conditions could reasonably represent an unmeasured slot, while the measurement library and schedule remained fixed (Table 2).

**Table 2:** Assignment rules and generated melanopic exposure summaries across 100,000 profiles per rule

Assignment rule	Eligible pool or matching rule	Median dose	Dose, 2.5th–97.5th pct	TAT > 250	TAT > 1000	TAT > 10,000
		(lx ⋅ h)	(lx ⋅ h)	(h)	(h)	(h)
Unrestricted pooled	All 63 observations for every slot	45,559	21,991–77,704	6.5	5.0	2.0
Outdoor-only bound	All 18 outdoor observations for every slot	119,995	91,585–155,817	11.0	11.0	6.5
Indoor-only bound	All 45 indoor observations for every slot	16,536	6,233–29,520	4.5	3.0	0.0
Exact-slot matched	Same 30-minute bin	44,257	30,693–60,497	6.5	5.0	2.0
Broad-context matched	Home, commute, office, or noon pool	48,337	30,252–73,254	6.5	5.5	2.0
Activity-block matched	Same morning, noon, or afternoon activity block	47,188	30,485–71,427	6.5	5.5	2.0

Melanopic EDI thresholds are reported in lx and dose in lx ⋅ h; TAT values are medians. These are descriptive outputs of the rules, not model-performance statistics. The number of distinct profiles each rule can generate is given in Methods.

The unrestricted pooled rule allowed any of the 63 measurements to fill any schedule slot. It retained only the number and order of the slots and ignored both clock time and environmental context. The indoor-only and outdoor-only rules were constructed as contrasting bounds. Under these rules, every slot was filled exclusively from the 45 indoor measurements or the 18 outdoor measurements, respectively. These bounds were included to show the consequences of making bright outdoor measurements either completely unavailable or available throughout the represented day.

The remaining three rules preserved different forms of temporal or contextual structure. Exact-slot matching allowed a slot to draw only from measurements collected in the same half-hour time bin and therefore retained clock time most directly. Broad-context matching grouped measurements into home, commute, office and noon categories, allowing values to move across clock times within the same category. Activity-block matching preserved the ordered sequence of morning home, morning commute, morning office, noon, afternoon office, afternoon commute and afternoon home, while allowing values to move among clock times within a block. These rules are alternatives rather than steps in a hierarchy: each retains a different feature of the route and embodies a different assumption about representativeness.

### Assignment rules changed profile spread more than median exposure

We held the 63-observation measurement library and the 22-slot schedule constant and changed only the eligible pool for each slot (Table 2).

**Fig. 2:**
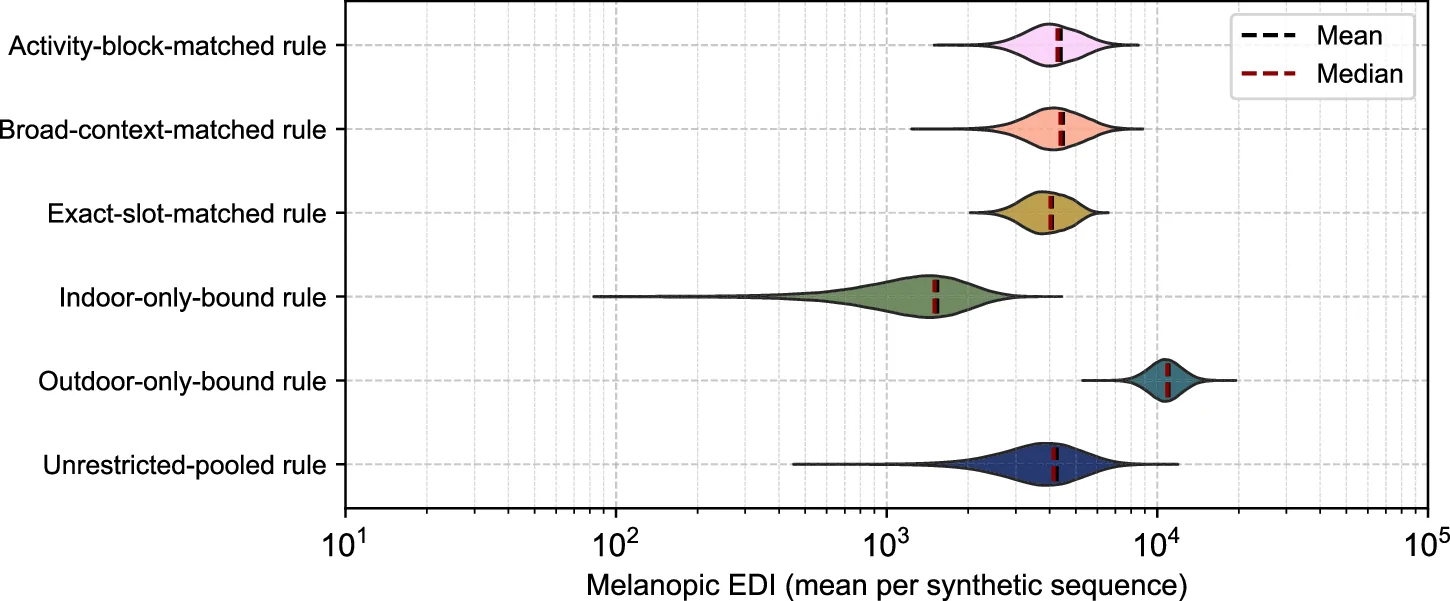
Mean melanopic EDI across profiles generated under six assignment rules. Each violin summarises 100,000 generated profiles constructed from the same 63-observation measurement library.

For each rule, we generated 100,000 profiles by sampling independently from the eligible measurement pool for every schedule slot. The deliberately extreme indoor-only and outdoor-only bounds produced the clearest contrast (Fig. 2 and Table 2). Median melanopic dose was 16,536 lx ⋅ h under the indoor-only bound and 119,995 lx ⋅ h under the outdoor-only bound. These distributions show what follows when the measured outdoor conditions are either excluded from every slot or made eligible throughout the represented day.

The four rules that combined indoor and outdoor measurements yielded similar median doses, from 44,257 to 48,337 lx ⋅ h, but markedly different widths. The central 95% of the dose distribution spanned 55,713 lx ⋅ h under unrestricted pooling and 29,804 lx ⋅ h under exact-slot matching, with broad-context and activity-block matching intermediate at 43,002 and 40,942 lx ⋅ h (Table 2). TAT behaved similarly: medians were close across the mixed-environment rules, whereas the tails depended on which measurements could represent each part of the schedule. In this measurement library, the assignment rule therefore changed the spread of constructed exposure more than its centre.

This division follows from the sampling scheme. Because slots are filled independently, the expected dose under any rule is Δ*t*∑_*i*_*μ*_*i*_, where *μ*_*i*_ is the mean of the pool eligible for slot *i*, and the expected TAT is the corresponding sum of exceedance probabilities. Central estimates are therefore governed by the composition of the eligible pools and by how many slots draw from each; resampling is informative about spread and tails rather than about the centre. The four mixed rules agree closely because their slot-weighted compositions are similar—broad-context matching, for example, assigns 0.27 of the slots to the commute pool, which supplies 0.24 of the library—so the small differences among their medians reflect these weightings rather than the structure each rule preserves. Generated medians fall slightly below the corresponding expected values because the dose distribution is right-skewed.

### Profiles generated under each rule

The rule-specific plots show how each eligibility constraint propagated through the daily profile. Every violin summarises 100,000 profiles assembled from the same 63 measurements. This large Monte Carlo sample reduces numerical noise in the displayed distributions, but it does not add independent measurement days.

#### Unrestricted pooled rule

**Fig. 3:**
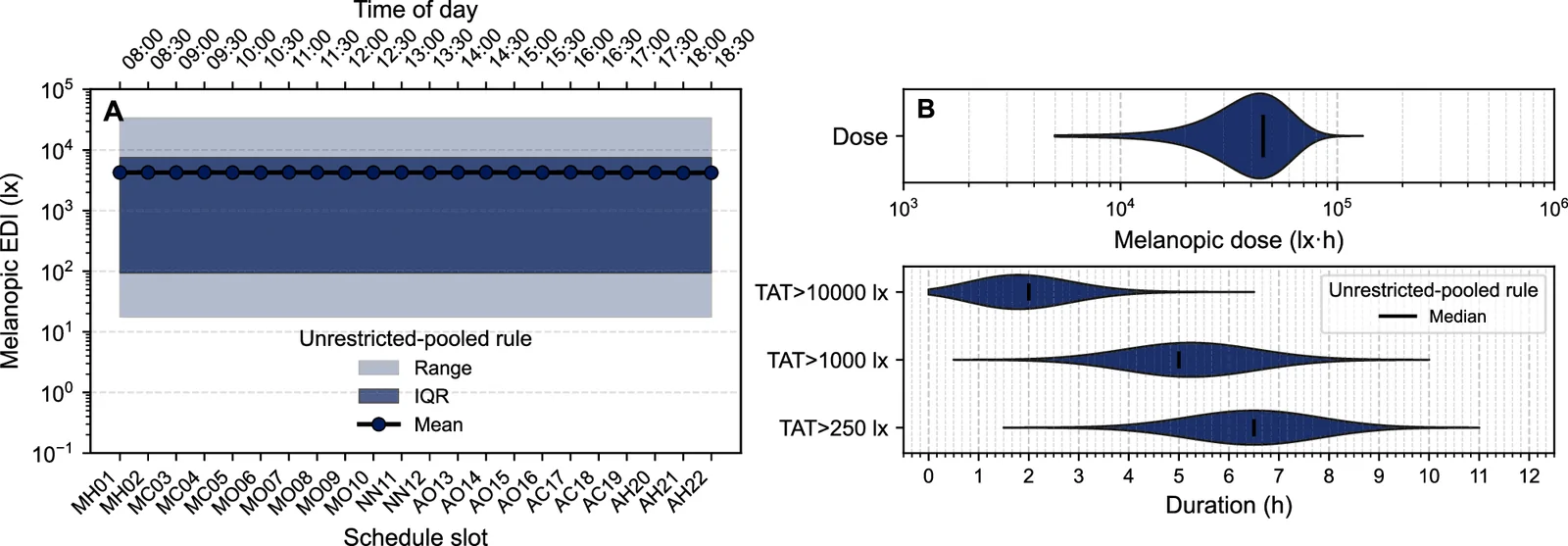
Melanopic exposure under the unrestricted pooled rule. **A** Generated-profile melanopic EDI across the 22 schedule slots (log scale). **B** Distributions of dose and TAT across generated profiles.

Under unrestricted pooling, any of the 63 measurements could fill any slot. The mean profile was therefore almost flat across the day, at ~4 × 10^3^ lx, and each slot had a broad spread (Fig. 3). Median dose was 45,559 lx ⋅ h (2.5th–97.5th percentiles: 21,991–77,704 lx ⋅ h). Median TAT was 6.5 h above 250 lx, 5 h above 1000 lx and 2 h above 10,000 lx. The similarity of these medians to the mean observed-day summaries is not evidence of accuracy. This rule deliberately removes the relation between time, activity context and exposure.

#### Outdoor-only bound

**Fig. 4:**
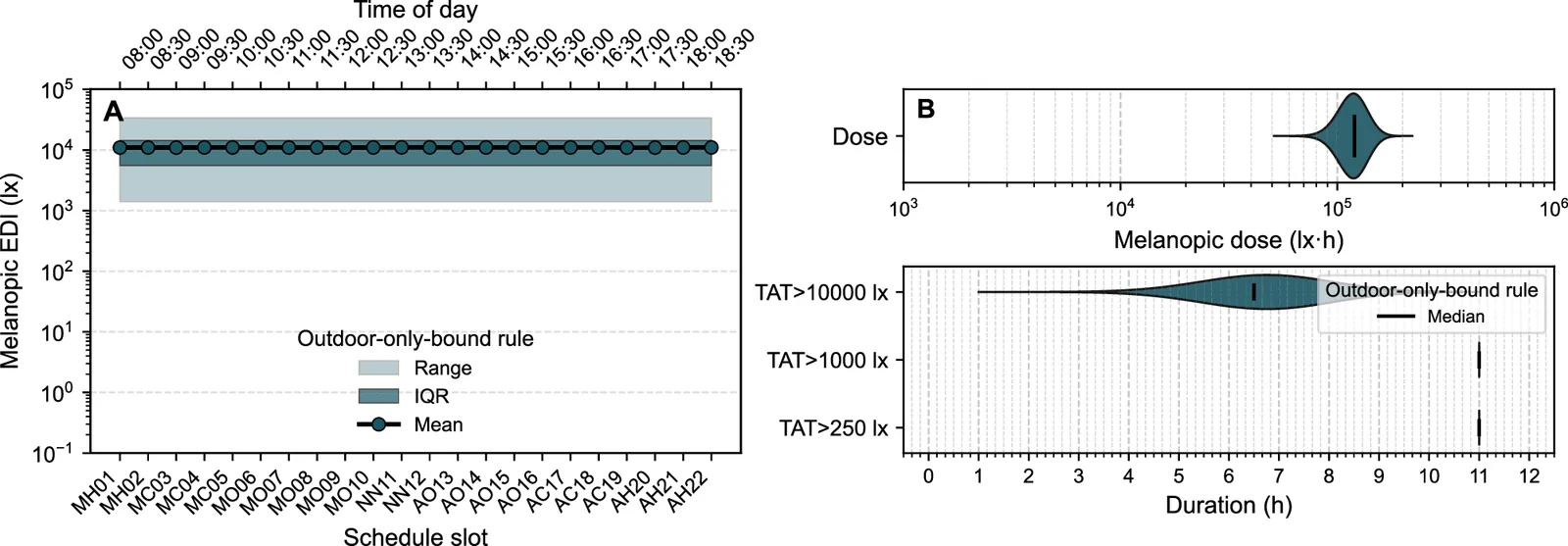
Melanopic exposure under the outdoor-only bound. **A** Generated-profile melanopic EDI across the 22 schedule slots (log scale). **B** Distributions of dose and TAT across generated profiles.

The outdoor-only bound allowed only the 18 outdoor measurements to fill all 22 slots. It consequently produced a high profile across the full represented day (Fig. 4). Median dose was 119,995 lx ⋅ h (2.5th–97.5th percentiles: 91,585–155,817 lx ⋅ h). Median TAT reached the full 11 h above both 250 and 1000 lx and was 6.5 h above 10,000 lx. This is not a prediction for a person spending a day outdoors, but the upper distribution generated when every slot is drawn from the measured outdoor pool.

#### Indoor-only bound

**Fig. 5:**
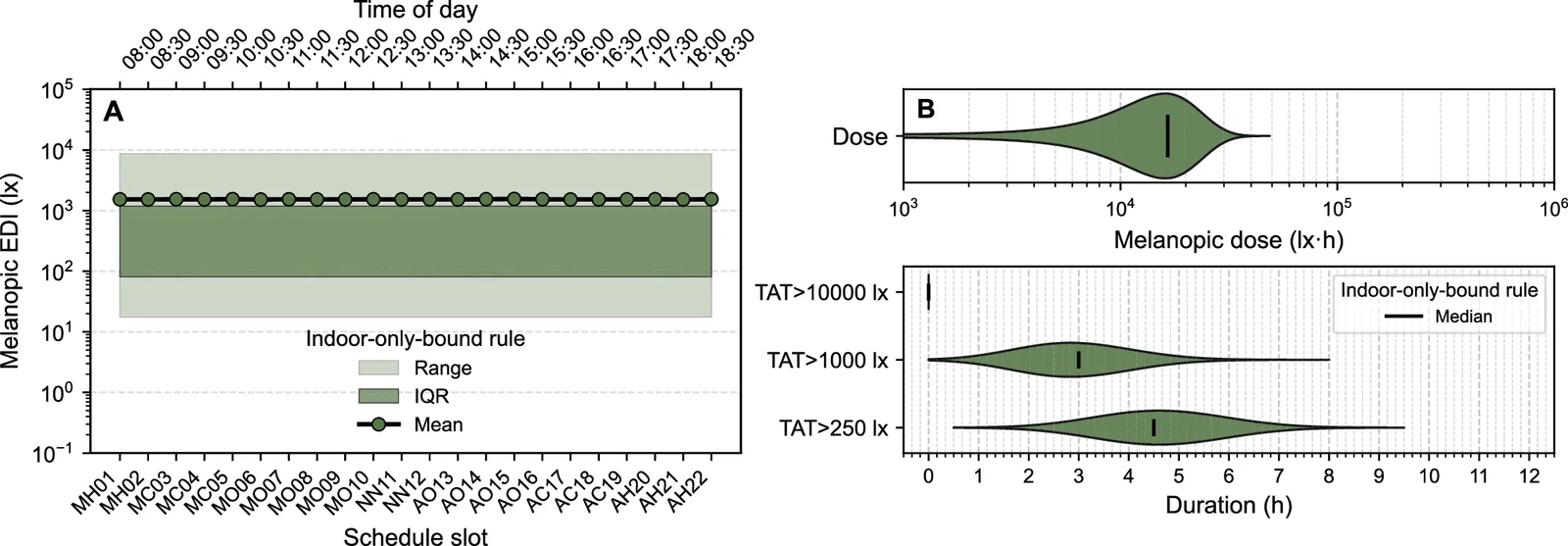
Melanopic exposure under the indoor-only bound. **A** Generated-profile melanopic EDI across the 22 schedule slots (log scale). **B** Distributions of dose and TAT across generated profiles.

The indoor-only bound allowed only the 45 indoor measurements to fill the schedule. The resulting profile was lower and more uniform than profiles that included outdoor measurements (Fig. 5). Median dose was 16,536 lx ⋅ h (2.5th–97.5th percentiles: 6233–29,520 lx ⋅ h). Median TAT was 4.5 h above 250 lx and 3 h above 1000 lx; median TAT above 10,000 lx was zero. As with the outdoor-only bound, this describes the consequences of an extreme assignment rule rather than a measured or predicted personal day.

#### Exact-slot-matched rule

**Fig. 6:**
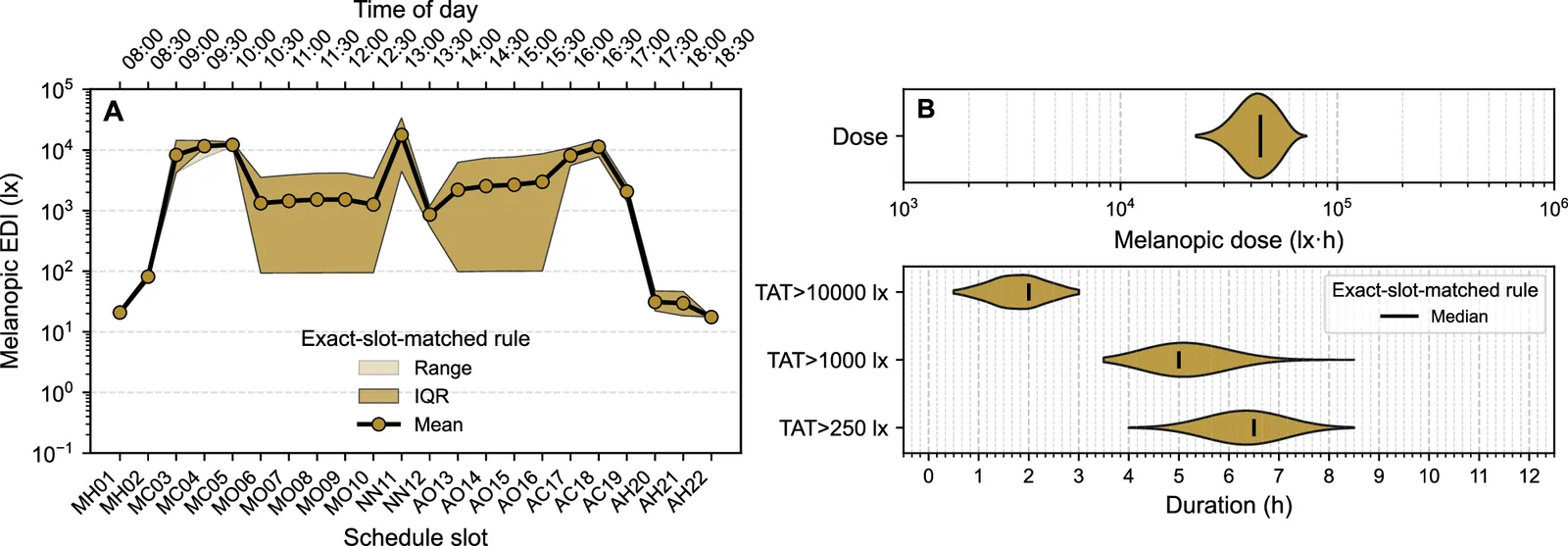
Melanopic exposure under the exact-slot-matched rule. **A** Generated-profile melanopic EDI across the 22 schedule slots (log scale). **B** Distributions of dose and TAT across generated profiles.

Exact-slot matching allowed a schedule slot to draw only from measurements recorded in the same half-hour bin. It therefore retained the clock-time pattern most directly, including outdoor peaks and lower indoor periods (Fig. 6). Median dose was 44,257 lx ⋅ h (2.5th–97.5th percentiles: 30,693–60,497 lx ⋅ h). Median TAT was 6.5 h above 250 lx, 5 h above 1000 lx and 2 h above 10,000 lx. This rule produced the narrowest dose span among the four mixed-environment rules because each slot drew from a small time-specific pool. The narrower span reflects the sampling design, not necessarily lower variability in daily life.

#### Broad-context-matched rule

**Fig. 7:**
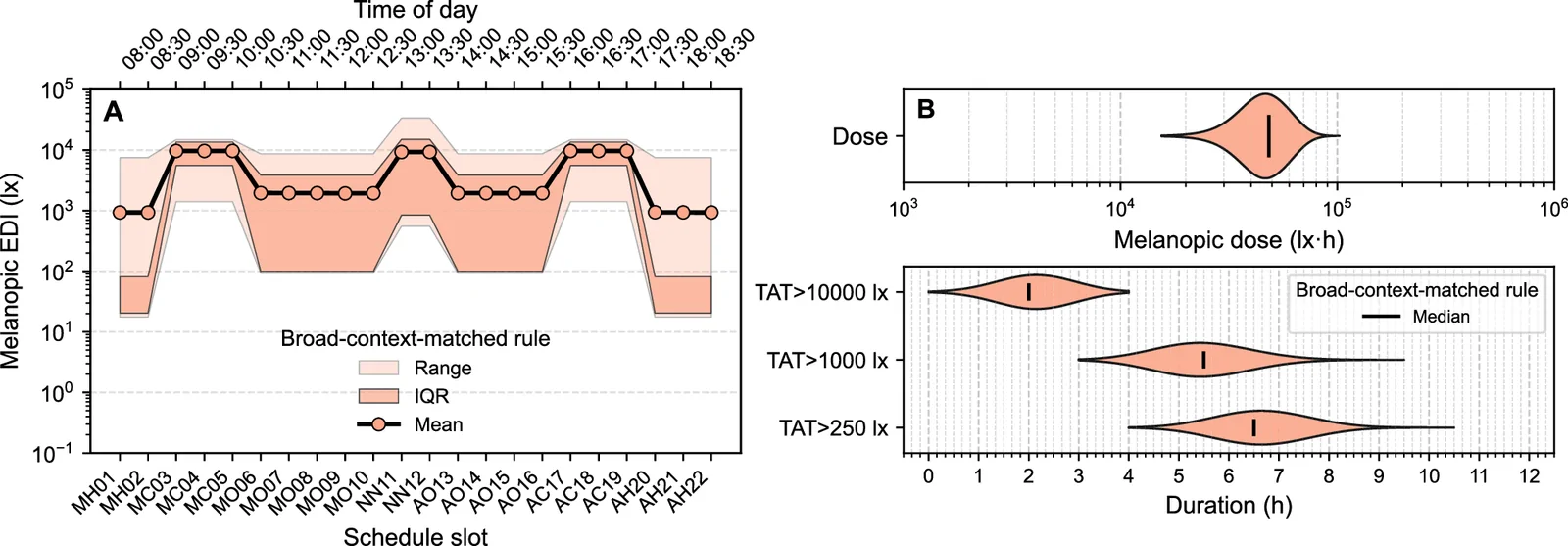
Melanopic exposure under the broad-context-matched rule. **A** Generated-profile melanopic EDI across the 22 schedule slots (log scale). **B** Distributions of dose and TAT across generated profiles.

Broad-context matching retained four categories: home, commute, office and noon. A slot could draw from any measurement in its category, irrespective of the exact clock time or whether it was recorded in the morning or afternoon. The resulting profile retained the broad sequence of contexts while allowing more movement of values within each category (Fig. 7). Median dose was 48,337 lx ⋅ h (2.5th–97.5th percentiles: 30,252–73,254 lx ⋅ h). Median TAT was 6.5 h above 250 lx, 5.5 h above 1000 lx and 2 h above 10,000 lx. Its larger eligible pools produced a wider distribution than exact-slot matching in this measurement library.

#### Activity-block-matched rule

**Fig. 8:**
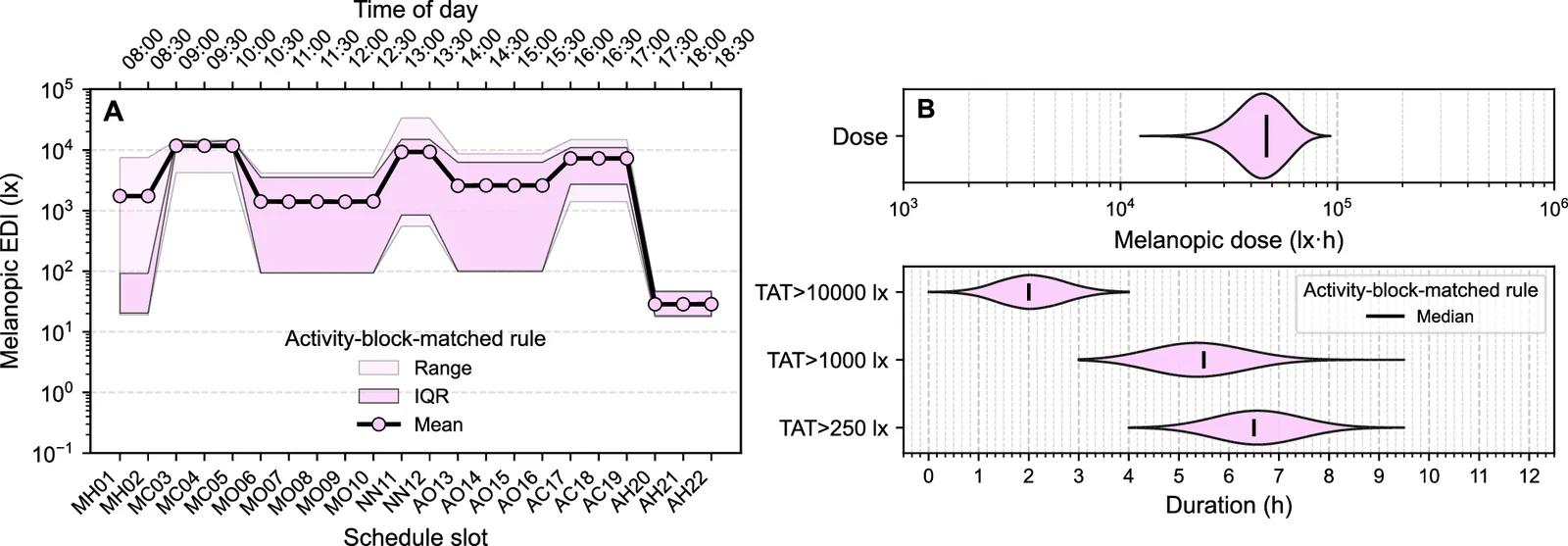
Melanopic exposure under the activity-block-matched rule. **A** Generated-profile melanopic EDI across the 22 schedule slots (log scale). **B** Distributions of dose and TAT across generated profiles.

Activity-block matching distinguished seven ordered blocks: morning home, morning commute, morning office, noon, afternoon office, afternoon commute and afternoon home. Measurements could move among clock times within a block but not between blocks. The generated profile therefore followed the route’s activity order while retaining variation within each block (Fig. 8). Median dose was 47,188 lx ⋅ h (2.5th–97.5th percentiles: 30,485–71,427 lx ⋅ h). Median TAT was 6.5 h above 250 lx, 5.5 h above 1000 lx and 2 h above 10,000 lx. This rule is distinct from exact-slot matching because it preserves activity-block membership rather than exact clock time.

### Commute and lunch substitutions affected dose and TAT differently

**Fig. 9:**
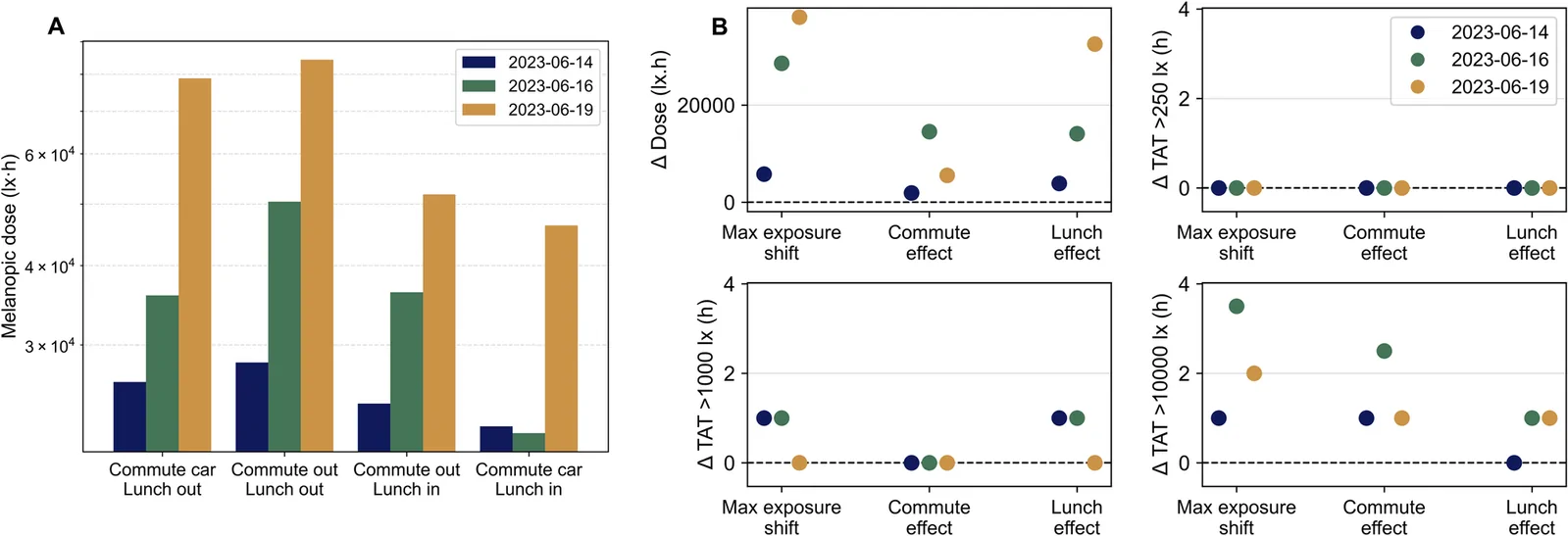
Melanopic exposure across targeted commute and lunch substitutions. **A** Absolute melanopic dose for four combinations of commute and lunch conditions on each measurement day. **B** Paired within-day differences in dose and TAT. The dashed line indicates no difference.

After comparing assignment rules, we changed only the commute and lunch conditions while leaving the home and office values for each measured day unchanged. The four combinations paired outdoor walking or car commuting with outdoor or indoor lunch. Moving from car commute with indoor lunch to outdoor commute with outdoor lunch produced the largest dose increase, with a median within-day difference of 28,600 lx ⋅ h (minimum–maximum: 5810–38,082 lx ⋅ h; positive on all three days). The lunch contrast had a larger median dose difference than the commute contrast (14,093 versus 5517 lx ⋅ h), although both were positive on all three days (Fig. 9 and Table 3). Within each day the commute and lunch contrasts sum exactly to the combined substitution; the medians in Table 3 do not, because the median is not a linear operator.

**Table 3:** Within-day differences for the targeted scenario substitutions across three measurement days

	Combined substitution			Commute contrast			Lunch contrast		
Δ summary	Median	Max	Positive days	Median	Max	Positive days	Median	Max	Positive days
Δ dose (lx ⋅ h)	28,600	38,082	3/3	5,517	14,506	3/3	14,093	32,565	3/3
Δ TAT > 250 lx (h)	0.0	0.0	0/3	0.0	0.0	0/3	0.0	0.0	0/3
Δ TAT > 1000 lx (h)	1.0	1.0	2/3	0.0	0.0	0/3	1.0	1.0	2/3
Δ TAT > 10,000 lx (h)	2.0	3.5	3/3	1.0	2.5	3/3	1.0	1.0	2/3

Values show the median and maximum difference and the number of days with a positive difference. Melanopic EDI thresholds are reported in lx and dose in lx ⋅ h.

For any exposure summary, let *S*(*c*, *ℓ*) denote the value under commute condition *c* ∈ {out, car} and lunch condition *ℓ* ∈ {out, in}. The contrasts were

1 $${\Delta }_{\max }=S({{\rm{out}}},{{\rm{out}}})-S({{\rm{car}}},{{\rm{in}}}),$$

2 $${\Delta }_{{{\rm{commute}}}}=\frac{[S({{\rm{out}}},{{\rm{out}}})-S({{\rm{car}}},{{\rm{out}}})]+[S({{\rm{out}}},{{\rm{in}}})-S({{\rm{car}}},{{\rm{in}}})]}{2},$$

3 $${\Delta }_{{{\rm{lunch}}}}=\frac{[S({{\rm{out}}},{{\rm{out}}})-S({{\rm{out}}},{{\rm{in}}})]+[S({{\rm{car}}},{{\rm{out}}})-S({{\rm{car}}},{{\rm{in}}})]}{2}.$$

Dose and the three TAT thresholds did not respond in the same way. TAT above 250 lx did not change because all affected epochs already exceeded that level in the compared conditions. For TAT above 1000 lx, the combined and lunch contrasts had median increases of 1 h and were positive on two of the three days, while the commute contrast was zero. Above 10,000 lx, the combined contrast increased TAT by a median of 2 h and was positive on all days. The commute contrast increased it by a median of 1 h on all days, whereas the lunch contrast increased it by a median of 1 h on two days. In this measurement library, outdoor lunch added more cumulative dose, while outdoor commute contributed more consistently to the highest-threshold duration. These case-specific patterns illustrate why dose and TAT are complementary rather than interchangeable summaries.

## Discussion

This study examined how strongly constructed melanopic light exposure summaries depend on the rule used to place sparse measurements into a daily schedule. In this measurement library, the clearest effect was on spread rather than on central exposure: the four rules that used both indoor and outdoor measurements produced median doses within a narrow range, yet their central 95% intervals differed in width by nearly a factor of two. That separation is a property of the sampling scheme as much as of these data. Independent slot-wise sampling fixes the expected dose at the slot-weighted sum of pool means, so the centre of a constructed profile follows from which measurements are eligible and how often they are drawn, and can be anticipated from the pools before any profiles are generated. What the rule contributes is the shape of the distribution around that centre. The distribution generated under each rule should therefore be read as a description of what that rule allows within this measurement library, not as a population distribution of personal exposure.

The indoor-only and outdoor-only assignments made the role of the measurement pool especially visible. Excluding all outdoor measurements produced the lowest exposure summaries, whereas allowing outdoor values in every slot produced the highest. These were intentionally artificial bounds rather than realistic personal schedules. They also expose a basic limitation of resampling: it can recombine measured conditions, but it cannot create a season, weather condition, location or behaviour that is absent from the source library. The numerical position of each bound would therefore change with a different route or set of environmental measurements.

The three structure-preserving rules retained different information. Exact-slot matching preserved clock time, broad-context matching preserved home, commute, office and noon categories, and activity-block matching preserved the ordered morning, noon and afternoon activity blocks. They are alternatives rather than steps in a single hierarchy. Exact-slot matching produced the narrowest distribution here because each time bin contained few eligible observations. This finding does not show that clock time is intrinsically the best predictor of exposure; a larger or differently balanced measurement library could change the relative spread produced by the rules.

The commute and lunch substitutions further showed why a single daily summary can conceal meaningful differences in temporal exposure. Outdoor lunch produced a larger increase in cumulative melanopic dose, consistent with the bright midday measurements, whereas outdoor commute contributed more consistently to time above 10,000 lx. Neither substitution changed time above 250 lx because the affected epochs already exceeded that threshold in the compared conditions. Dose and TAT therefore captured different features of the same local substitutions. These comparisons remain scenario analyses rather than causal estimates: no participant was observed or assigned to a commute or lunch condition, and the scenarios reused a small set of environmental measurements.

Continuous wearable measurement remains the more direct method when the aim is to characterise an individual’s experienced light exposure. The present framework has a narrower role: it is useful when calibrated measurements have been collected at selected route points and the investigator needs to understand how explicit assumptions about time and context shape a constructed profile. Potential applications include environmental protocols, scenario comparisons and study planning. The method cannot recover compliance, gaze direction, body shading, unsampled activities or exposure between measured epochs, and it should not be presented as a substitute for continuous personal dosimetry.

We did not use inferential tests to compare assignment rules because the 100,000 profiles generated under each rule are repeated transformations of the same 63 observations, not independent empirical days. Treating them as independent would greatly overstate the empirical sample size of three measurement days. The convergence analysis establishes numerical stability for the implemented Monte Carlo procedure only; it does not validate the profiles against personal exposure or increase their generalisability.

Several features of the study constrain interpretation. Measurements were collected along one route, in one city and season, on three non-consecutive days. The planned 22-slot route was not measured completely on every date, and one date included an additional alternative commute observation, so observed-day summaries covered unequal durations. Sampling with replacement ignores serial dependence within a measured day and can combine values that were never observed together; because real exposure sequences are positively autocorrelated, the generated spreads should be read as lower bounds on the variability of comparable real days. The represented window ran from 08:00 to 18:30 and therefore excluded early-morning and evening light. View direction was recorded as contextual metadata but not varied in the analysis, although viewing geometry is itself a substantial source of variation in eye-level illuminance. The analysis did not quantify instrument uncertainty and lacked simultaneous continuous eye-level or wearable measurements for validation. Increasing the number of generated profiles cannot remove these limitations.

The practical lesson is procedural. Investigators who use sparse environmental measurements to represent a daily schedule should state the schedule slots and eligible pools explicitly, report the composition of those pools, distinguish measured from generated durations, and show sensitivity to reasonable alternative rules. In this three-day example, those choices changed the spread of melanopic exposure summaries more than their central values. Reporting the assignment step is what allows a reader to judge what a constructed light exposure profile represents and what it does not.

## Methods

### Study design and measurement route

This environmental measurement study enrolled no participants and used no human data. Measurements were collected in Ottawa, Canada, on 14, 16 and 19 June 2023. The predefined route followed seven ordered activity blocks: morning home (MH), morning commute (MC), morning office (MO), noon or lunch (NN), afternoon office (AO), afternoon commute (AC) and afternoon home (AH). The schedule contained 22 half-hour slots with start times from 08:00 through 18:30, spanning 11 h. Measurements were obtained at planned times where possible, with additional observations of alternative commute or lunch conditions and some planned slots not observed. The final library contained 23 observations on 14 June, 21 on 16 June and 19 on 19 June, for a total of 63. Forty-five observations were classified as indoor and 18 as outdoor (Table 4).

**Table 4:** Number of spectral irradiance observations collected on each daily-route measurement day

Date	Indoor observations	Outdoor observations	Total observations
14 June 2023	16	7	23
16 June 2023	14	7	21
19 June 2023	15	4	19
Total	45	18	63

At each observation, eye-level spectral irradiance was measured with a high-resolution spectroradiometer fitted with a cosine diffuser (spectraval 1511-HiRes, JETI Technische Instrumente GmbH, Jena, Germany; 380–780 nm, 1-nm spectral sampling). The instrument was positioned ~120 cm above floor level and oriented vertically to approximate light incident at eye level. Wide-angle RGB images were captured with an uncalibrated GoPro HERO10 Black camera, and contextual metadata were recorded, including date, time, indoor or outdoor classification, scene category, view category, weather and ambient temperature. Additional modalities were collected but were not used in this analysis. Workplace measurements came from three offices at the National Research Council campus: one with electric lighting only and two with daylight and electric lighting. Outdoor measurements were collected along the ~2-km route and on the campus. Commute measurements represented outdoor walking and in-car conditions; lunch measurements represented an indoor kitchen and an outdoor condition. A rooftop spectroradiometer measured downwelling photopic illuminance at one-minute resolution as a daylight reference for Fig. 1.

### Melanopic exposure metrics and represented time

Spectral irradiance was converted to photopic illuminance and melanopic EDI according to CIE S 026^6^. Throughout, melanopic EDI is reported in lx and melanopic dose in lx ⋅ h; threshold labels in lx refer to melanopic EDI. Melanopic dose and TAT were computed using LightLogR and the same half-hour convention throughout the supplied analysis code^19^. Each measured or assigned value represented one 30-min epoch. If *x*_*i*_ is the melanopic EDI assigned to epoch *i* and Δ*t* = 0.5 h, dose for a profile with *m* epochs was

4 $$D=\Delta t{\sum}_{i = 1}^{m}{x}_{i},$$

and TAT above threshold *τ* was

5 $${{{\rm{TAT}}}}_{\tau }=\Delta t{\sum }_{i = 1}^{m}{\mathbb{I}}({x}_{i} > \tau ),$$

where ${\mathbb{I}}$ equals one when the epoch value exceeds the threshold and zero otherwise. No interpolation was used. Generated profiles and targeted scenarios contained 22 epochs and represented 11 h. The observed-day summaries used every available observation on each date and therefore represented 11.5, 10.5 and 9.5 h. No analysed value was exactly equal to 250, 1000 or 10,000 lx, so using a strict rather than inclusive threshold did not alter the results.

TAT was evaluated at melanopic EDI thresholds of 250, 1000 and 10,000 lx. The 250-lx level corresponds to an expert daytime recommendation for healthy adults^7^. The 1000-lx level provided an intermediate marker often reached in daylight-rich conditions, and 10,000 lx was used as a descriptive marker of high daylight exposure. The latter two levels were not treated as physiological recommendations.

### Constructing daily profiles from sparse measurements

**Fig. 10:**
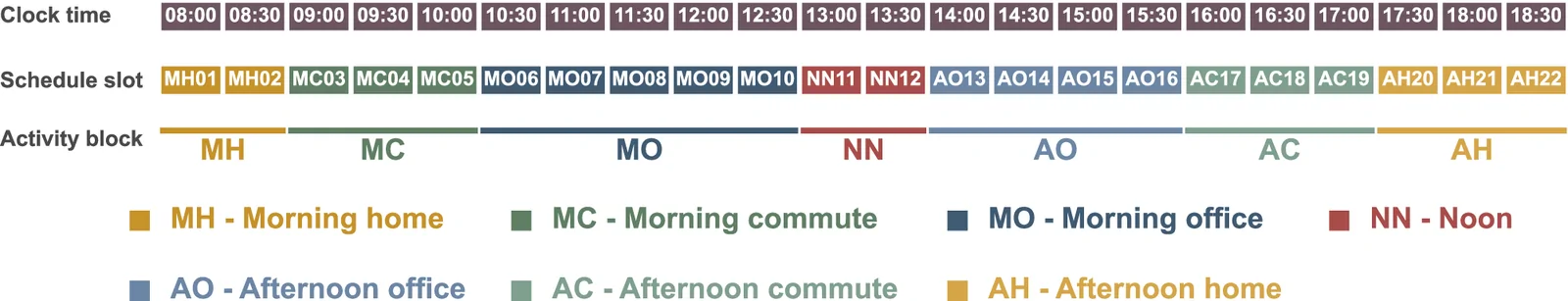
The planned daily-route schedule. The route contains 22 ordered 30-minute schedule slots grouped into morning home (MH), morning commute (MC), morning office (MO), noon or lunch (NN), afternoon office (AO), afternoon commute (AC) and afternoon home (AH) blocks.

A generated profile was an ordered vector of 22 melanopic EDI values, one for each half-hour schedule slot shown in Fig. 10. An assignment rule determined which of the 63 observed values were eligible for each slot; Table 5 states the structure retained and ignored by each rule. Values were then sampled with replacement from the eligible pool, independently for each slot. For example, the 14:00 slot could draw from any measurement under unrestricted pooling, only measurements from the 14:00 bin under exact-slot matching, any office measurement under broad-context matching, or any afternoon-office measurement under activity-block matching. The output was conditional on the measured values, fixed schedule and assignment rule; it was not a reconstruction of a continuously observed day.

**Table 5:** Definitions of the six measurement-assignment rules

Rule	Eligible observations for each slot	Retained structure	Ignored structure
Unrestricted pooled	Any of the 63 observations	Number and order of slots	Clock time and context
Outdoor-only bound	Any of the 18 outdoor observations	Number and order of slots; outdoor condition	Clock time and activity context
Indoor-only bound	Any of the 45 indoor observations	Number and order of slots; indoor condition	Clock time and activity context
Exact-slot matched	Observations from the same half-hour bin	Clock time	Context within a time bin
Broad-context matched	Observations from the same home, commute, office, or noon pool	Broad daily context	Morning or afternoon distinction and exact time
Activity-block matched	Observations from the same seven-part activity block	Ordered activity block	Exact clock time within a block

The rules preserve different aspects of the schedule and are not presented as a nested hierarchy.

**Fig. 11:**
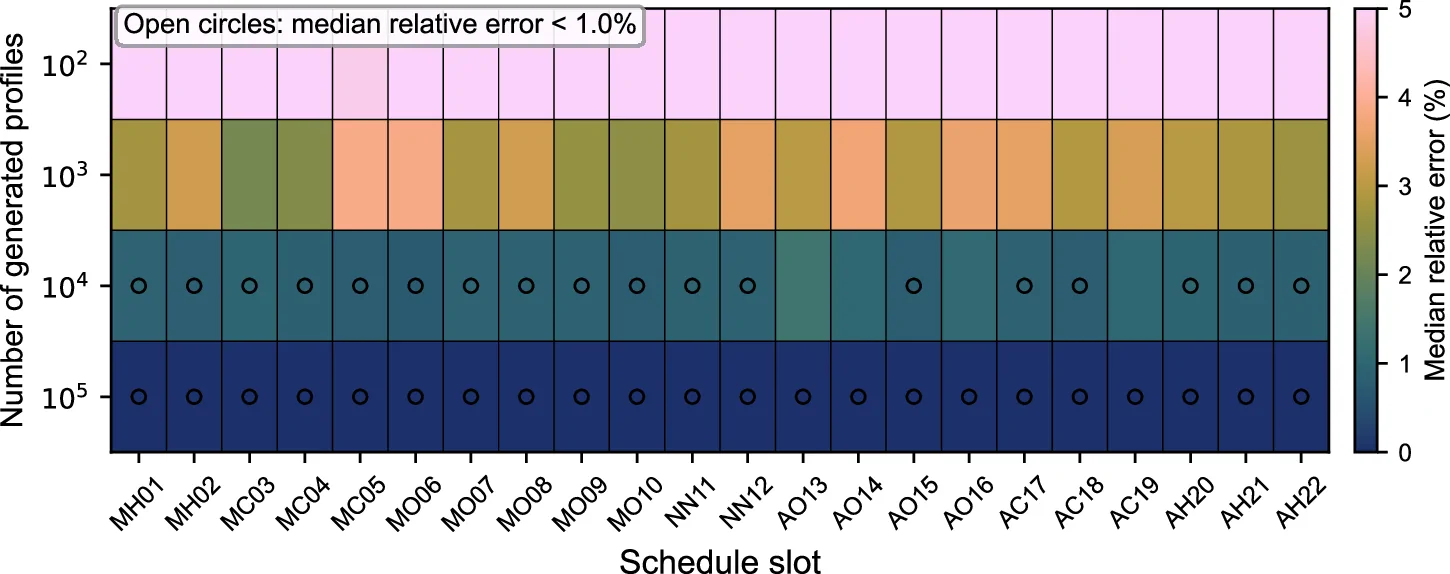
Monte Carlo convergence analysis for the unrestricted pooled rule. The heatmap shows the median relative error (%) of the mean melanopic EDI estimate at each 30-min schedule slot for sample sizes from 10^2^ to 10^5^, computed over 50 repetitions. At 10^4^ profiles, most slots had a median relative error below 1%; at 10^5^, all slots met the predefined criterion. We therefore used 10^5^ profiles for resampling.

For each rule, 100,000 profiles were generated. The possible profile spaces were too large for exhaustive enumeration, so Monte Carlo sampling was used to describe the resulting distributions^20,21^. Numerical stability was examined for the unrestricted pooled rule at 10^2^, 10^3^, 10^4^ and 10^5^ profiles. For each sample size, the mean melanopic EDI at every slot was compared with the estimate from the largest sample across 50 repetitions. The predefined stability criterion was a median relative error below 1% at every schedule slot. Relative error decreased with sample size, and 100,000 profiles met this criterion (Fig. 11). This check addressed Monte Carlo error only, not measurement uncertainty or validity against unobserved personal exposure.

### Implementation of the assignment rules

Under the unrestricted pooled rule, all 63 observations were eligible for every slot, yielding ~3.9 × 10^39^ possible ordered profiles. The indoor-only and outdoor-only bounds used the 45 indoor or 18 outdoor observations, respectively, for every slot, yielding ~2.3 × 10^36^ and 4.1 × 10^27^ profiles. These three rules retained the length and order of the schedule but ignored clock time and activity context. All such counts describe the size of the space a rule can generate; they are not sample sizes, and the empirical sample remains three measurement days.

Under exact-slot matching, an observation was eligible only for the corresponding half-hour bin. Eligible pool sizes across the 22 slots ranged from one to four observations. Their product yielded ~6.2 × 10^9^ possible ordered profiles. Context was not separately restricted within a time bin.

For broad-context matching, observations were grouped into home, commute, office and noon pools. Morning and afternoon observations were combined within the home, commute and office categories. The implemented pools contained 15 home-classified, 15 commute-classified, 27 office-classified and 6 noon observations. Five home slots, six commute slots, nine office slots and two noon slots drew from their corresponding pools, yielding ~2.4 × 10^27^ possible profiles.

For activity-block matching, observations remained within seven ordered activity blocks. The eligible pools contained 8 morning-home, 8 morning-commute, 15 morning-office, 6 noon, 12 afternoon-office, 7 afternoon-commute and 7 afternoon-home observations. The schedule contained 2, 3, 5, 2, 4, 3 and 3 slots in those blocks, respectively, yielding ~2.2 × 10^21^ possible profiles. Values could move across clock times within a block but not between blocks.

### Targeted commute and lunch substitutions

**Fig. 12:**
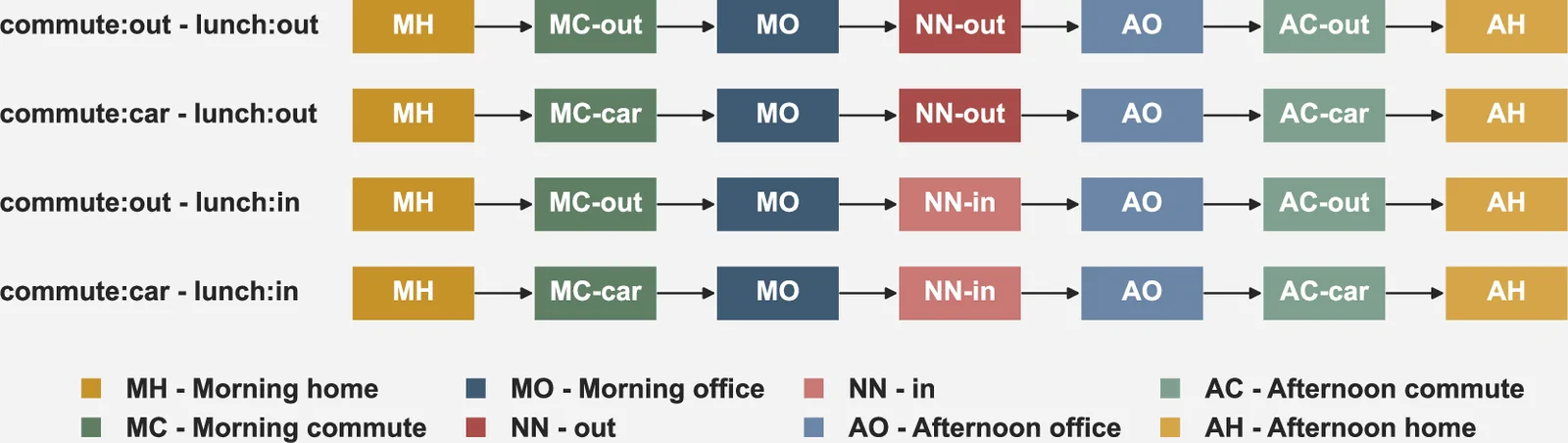
Targeted scenario substitutions derived from the same base day. Only commute mode and lunch condition differ among the four profiles; home and office blocks remain fixed.

Each observed day was also used as a fixed base profile in which only commute and lunch values were changed. Home and office values remained unchanged. Four scenarios combined outdoor walking or car commuting with outdoor or indoor lunch (Fig. 12). Outdoor commute and lunch values came from the corresponding observed conditions. Because only three in-car measurements were available, all in-car slots used the mean melanopic EDI of those measurements to represent a common car condition. Dose and TAT were recalculated using the 0.5-h epoch convention. The paired contrasts compared the combined substitution, the average commute difference across lunch conditions and the average lunch difference across commute conditions.

### Statistics and reproducibility

The empirical sample comprised three independent measurement days and 63 environmental spectral irradiance observations. Results for the measured days are reported by date, with the arithmetic mean provided as a descriptive cross-day summary. Generated-profile results are reported as medians with 2.5th–97.5th percentiles across 100,000 profiles per assignment rule. Targeted scenario differences are reported as medians, maxima and the number of days with a positive difference. No hypothesis tests or *P* values were calculated. The generated profiles are dependent resamples of the same measurement library and were not treated as independent observations for inferential testing. The data-processing and profile-generation code is available in the repository listed below.

To assess the computational reproducibility of the study, the analysis workflow was evaluated through CODECHECK, an independent process in which a checker executes the archived code and verifies whether the specified computational results can be reproduced from the deposited data and supporting materials^22^. The check covered the generation of the reported numerical summaries and figures from the archived computational workflow. Following successful completion of this independent check, a CODECHECK certificate was issued and publicly archived (CODECHECK certificate 2026-025)^23^.

## Data Availability

The datasets generated and analysed in this study are available in the GitHub repository (https://github.com/tscnlab/TabandehEtAl_npjBiolTimingSleep_2026) and archived on Zenodo (https://doi.org/10.5281/zenodo.21558893).

## Code availability

The code used to process the measurements, generate the profiles and reproduce the reported summaries and figures is available in the GitHub repository (https://github.com/tscnlab/TabandehEtAl_npjBiolTimingSleep_2026) and archived on Zenodo https://doi.org/10.5281/zenodo.21558893. The computational workflow was independently evaluated through CODECHECK, and a certificate documenting the scope and outcome of the check was issued and publicly archived on Zenodo (https://doi.org/10.5281/zenodo.22935939).
